# Isolation and propagation of leptospires at 37 °C directly from the mammalian host

**DOI:** 10.1038/s41598-020-66526-4

**Published:** 2020-06-15

**Authors:** Richard L. Hornsby, David P. Alt, Jarlath E. Nally

**Affiliations:** Infectious Bacterial Diseases Research Unit, National Animal Disease Center, Agricultural Research Service, United States Department of Agriculture, Ames, IA USA

**Keywords:** Microbiology techniques, Bacterial techniques and applications, Bacteriology, Microbiology

## Abstract

The causative agent of leptospirosis includes multiple serovars and species of pathogenic leptospires that are excreted via urine from reservoir hosts of infection. Primary isolation takes weeks to months, and is limited to semi-solid media at 28–30 °C. Here we present an alternative media formulation, HAN, compared to commercially available EMJH and the more specialized T80/40/LH media formulations, in semi-solid and liquid compositions, for the primary isolation of two diverse species and serovars of pathogenic leptospires directly from host kidney tissue. All three media types supported the isolation and propagation of *L*. *interrogans* serovar Copenhageni strain IC:20:001 in semi-solid media at 29 °C. However, only HAN and T80/40/LH supported the growth of *L*. *borgpetersenii* serovar Hardjo strain HB15B203 at 29 °C. In addition, HAN supported primary isolation at 37 °C. Both T80/40/LH and HAN supported primary isolation of strain IC:20:001 in liquid media at 29 °C but only HAN supported growth of strain HB15B203 in liquid media, at both 29 and 37 °C. HAN media supports the primary isolation of fastidious pathogenic leptospires directly from infected host tissue at either 29 or 37 °C: this formulation represents a more defined media for the continued optimization of growth factors required to support the primary isolation of the large and diverse range of species and serovars within the genus *Leptospira* circulating within domestic and wild animal populations.

## Introduction

Leptospirosis is a global zoonotic disease caused by pathogenic species of *Leptospira*^1^. Reservoir hosts, including domestic and wild animal species, excrete leptospires from colonized renal tubules via urine into the environment where they can persist in suitable moist conditions. Contact with contaminated environments, or directly with urine from reservoir hosts, can result in infection in incidental hosts that can range from a mild fever to the more severe icteric disease and massive pulmonary hemorrhage^2^. Over 1 million people suffer acute leptospirosis each year, with an estimated 58,900 deaths^3^.

Severe icteric leptospirosis was first described in 1886 by Adolph Weil^4^ but it was almost 30 years before the causative agent was identified by Inada *et al*.^5^. Subsequently, the significance of rats acting as reservoir hosts was established^6,7^, and with time, many domestic and wild animals were recognized as asymptomatic carriers of leptospires^2,8–10^. Historically, all pathogenic leptospires were classified as *L*. *interrogans* (sensu lato) while saprophytes were classified as *L*. *biflexa*, with both species comprising hundreds of serovars which were designated using a serologically based classification system. With the advent of genome sequencing, the genus *Leptospira* now comprises 64 pathogenic and saprophytic species^11^. The fastidious nature of pathogenic leptospires makes their primary isolation and propagation from animal species a laborious and time consuming task which was originally accomplished using specialized media containing undefined components including animal tissue, peptone, beef extracts and sera, and as pioneered by many investigators including Noguchi, Stuart, Korthoff, and Fletcher^2^. The use of a more defined media was advanced by Ellinghausen and McCullough who developed a serum free medium containing an oleic albumin complex (AOC) that supported the growth of 13 serotypes of leptospires^12^. AOC provided nitrogen and sulfur metabolites and was hypothesized to be effective due to its ability to bind oleic acid and other materials. In the absence of sera, AOC required the addition of vitamin B_12_. Fractionation of AOC determined that its growth supporting functions could be replaced by fraction V albumins, with water soluble polysorbate 80 as an oleate replacement^13^. The inclusion of albumin was hypothesized to detoxify fatty acids in growth media since the binding of fatty acids by serum albumin was a protective factor for the growth of tubercle bacilli^14^. Ellinghausen McCullough media was further modified by Johnson and Harris^15^, giving rise to EMJH, which is available commercially and routinely used as a media for the isolation and propagation of pathogenic and saprophytic leptospires worldwide.

It is axiomatic, but the reliance on a single media type to culture leptospires from mammalian hosts is selective for only those pathogenic leptospires capable of surviving in, or adapting to, the growth media and conditions used. Some pathogenic serovars require additional serum or other supplements, as exemplified by Ellis who formulated T80/40/LH to include polysorbate 40, lactalbumin hydrolysate, superoxide dismutase and rabbit serum, as required for the primary isolation and propagation of serovar Hardjo from cattle^16^. The primary isolation of leptospires from reservoir and incidental hosts is essential to understand pathogenic mechanisms of leptospirosis and mitigate disease transmission. Cultured isolates provide a definitive diagnosis, a strain for serotyping and epidemiological studies, a relevant serovar for inclusion in bacterin based vaccines to immunize animal species, and appropriate antigen for inclusion in microscopic agglutination test (MAT) panels and other diagnostic tests. In our current work, we present a novel media formulation, Hornsby-Alt-Nally (HAN), for the isolation and propagation of fastidious pathogenic leptospires directly from host tissue, and compare its effectiveness to EMJH and T80/40/LH, in both liquid and semi-solid media, at both 29 and 37 °C.

## Results

### Serology

All experimentally infected hamsters were seropositive at day 21 post-infection for the corresponding strain of infection, Table 1.

**Table 1:** MAT titers of experimentally infected hamsters.

Animal ID	Infecting strain	MAT with HB15B203	MAT with IC:20:001
1	IC:20:001	neg	50
2	IC:20:001	neg	100
3	IC:20:001	neg	50
4	IC:20:001	neg	50
5	HB15B203	400	neg
6	HB15B203	400	neg
7	HB15B203	200	neg
8	HB15B203	200	neg

Reciprocal titer of MAT with strain IC:20:001 or HB15B203 is reported. Neg = negative at 1:25 or greater.

### Semi-solid cultures

All experimentally infected hamsters were kidney culture positive 21 days after inoculation, Tables 2 and 3.

**Table 2:** Growth of kidney derived cultures of IC:20:001 and HB15B203 in semi-solid media at 29 °C.

Strain	Medium	PID 3	PID 7	PIW 2	PIW 3	PIW 4
IC:20:001	EMJH	—	—	D	D	D
IC:20:001	T80/40/LH	—	D	D	D	D
IC:20:001	HAN	—	D	D	D	D
HB15B203	EMJH	—	—	—	—	—
HB15B203	T80/40/LH	—	—	D	D	D
HB15B203	HAN	—	+ (6/12)	D	D	D

*L*. *interrogans* strain IC:20:001 and *L*. *borgpetersenii* strain HB15B203 were inoculated into EMJH, T80/40/LH or HAN semi-solid media incubated at 29 °C. Cultures were visually assessed for growth at post-inoculation day (PID) 3 and 7, and post-inoculation week (PIW) 2, 3 and 4. Cultures were scored as having no visible growth (-), visible growth confirmed by dark-field microscopy **(+**) or a visible Dinger zone (D) confirmed by dark-field microscopy. Results are derived from three replicates of kidney culture from four hamsters (N = 12) unless indicated in parentheses.

**Table 3:** Growth of kidney derived cultures of IC:20:001 and HB15B203 in semi-solid media at 37 °C.

Strain	Medium	PID 3	PID 7	PIW 2	PIW 3	PIW 4
IC:20:001	EMJH	—	—	—	—	—
IC:20:001	T80/40/LH	—	—	—	+ (6/12)	+ (6/12
IC:20:001	HAN	—	D	D	D	D
HB15B203	EMJH	—	—	—	—	—
HB15B203	T80/40/LH	—	—	—	—	—
HB15B203	HAN	—	+ (3/12)	D	D	D

*L*. *interrogans* strain IC:20:001 and *L*. *borgpetersenii* strain HB15B203 were inoculated into EMJH, T80/40/LH or HAN semi-solid media incubated at 37 °C. Cultures were visually assessed for growth at post-inoculation day (PID) 3 and 7, and post-inoculation week (PIW) 2, 3 and 4. Cultures were scored as having no visible growth (-), visible growth confirmed by dark-field microscopy (+) or a visible Dinger zone (D) confirmed by dark-field microscopy. Results are derived from three replicates of kidney culture from four hamsters (N = 12) unless indicated in parentheses.

**Figure 1:**
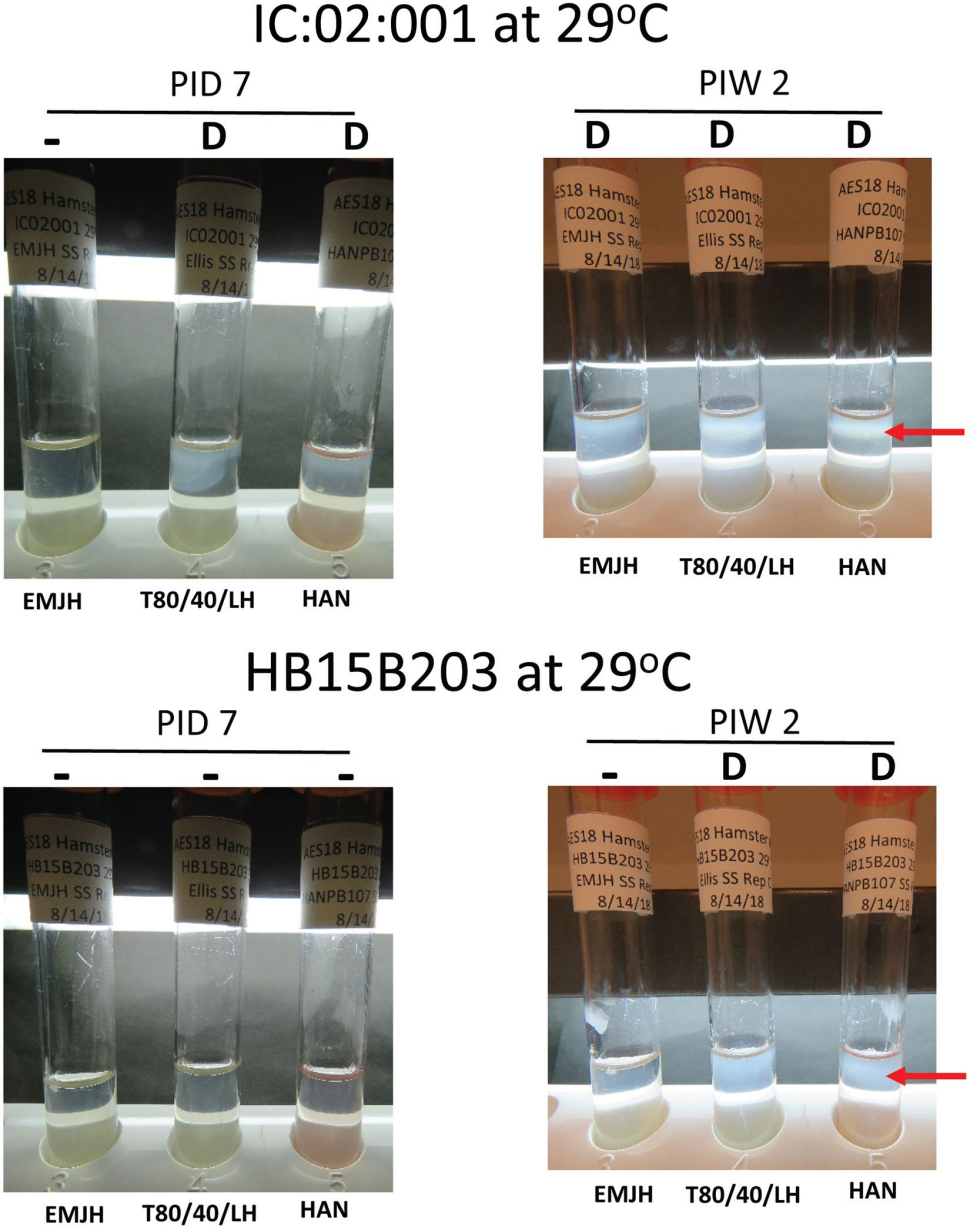
Representative images of EMJH, T80/40/LH and HAN semi-solid media incubated at 29 °C after inoculation with IC:20:001 or HB15B203. Growth in semi-solid media was assessed visually and scored as having no visible growth (-), visible growth (+) which was confirmed by dark-field microscopy or the presence of a Dinger zone (D) which was confirmed by dark-field microscopy. Red arrows indicate representative Dinger zones.

When incubated at 29 °C, strain IC:20:001 was promptly cultured in EMJH, T80/40/LH and HAN semi-solid media. Evidence of a Dinger zone was observed in T80/40/LH and HAN semi-solid media within 7 days post-inoculation, Table 2 and Fig. 1, with all three media showing the presence of a Dinger Zone by 2 weeks post-inoculation.

When incubated at 29 °C, strain HB15B203 was promptly cultured in both T80/40/LH and HAN semi-solid media by 2 weeks post-inoculation as evidenced by the presence of a Dinger zone. Evidence of visible growth was detected in 6 of 12 replicates in HAN semi-solid media within 7 days post-inoculation, and as confirmed by dark-field microscopy. Strain HB15B203 was not detected by 4 weeks post-inoculation in EMJH semi-solid media, Table 2 and Fig. 1.

**Figure 2:**
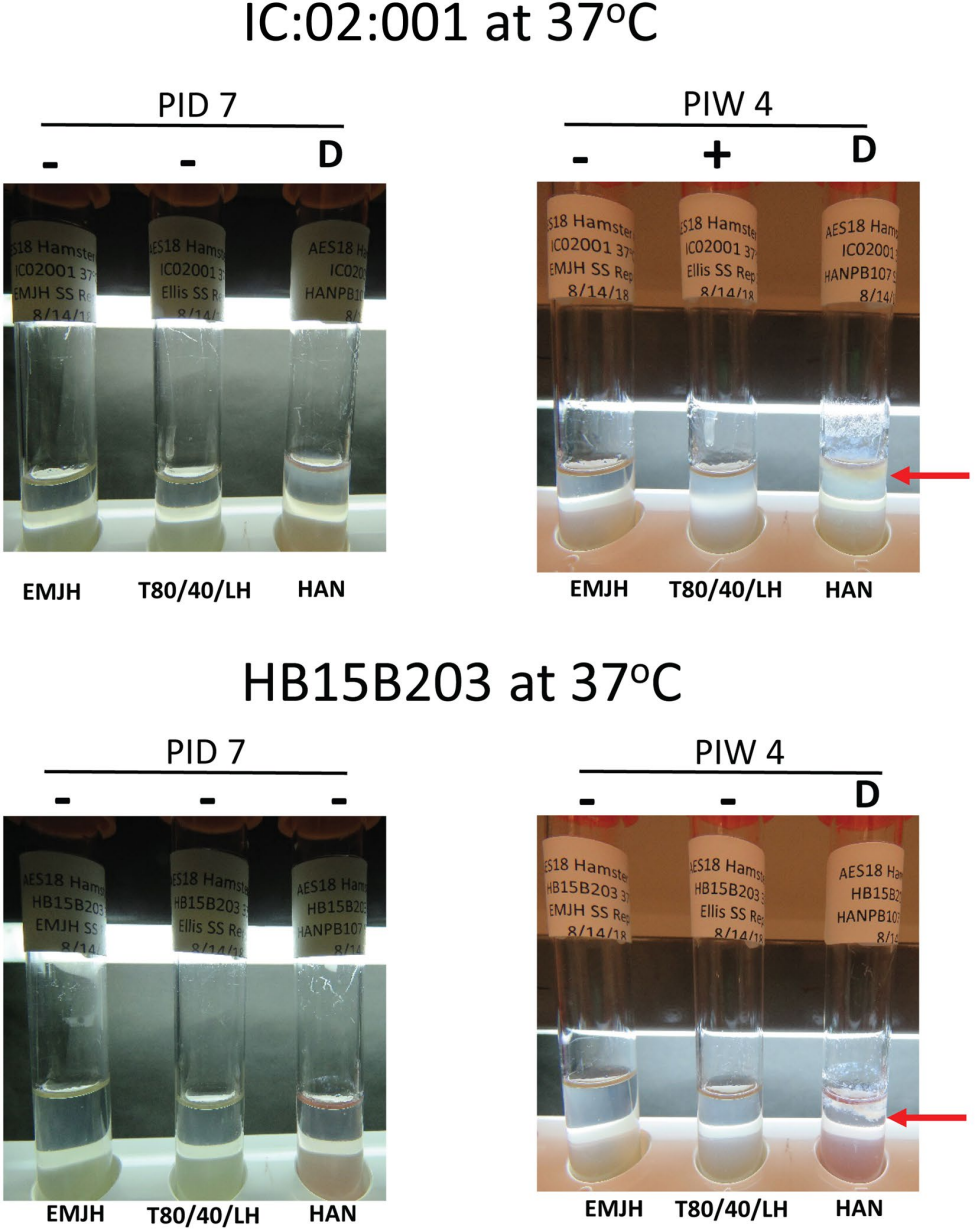
Representative images of EMJH, T80/40/LH and HAN semi-solid media incubated at 37 °C after inoculation with IC:20:001 or HB15B203. Growth in semi-solid media was assessed visually and scored as having no visible growth (-), visible growth (+) which was confirmed by dark-field microscopy or the presence of a Dinger zone (D) which was confirmed by dark-field microscopy. Red arrows indicate representative Dinger zones.

When incubated at 37 °C, strain IC:20:001 was only recovered in T80/40/LH and HAN semi-solid media. Six of 12 replicates were positive for growth in T80/40/LH by 3 weeks post-inoculation but Dinger zones were not observed. In contrast, Dinger zones were observed for IC:20:001 cultured in HAN semi-solid by 7 days post-inoculation, Table 3 and Fig. 2. Strain IC:20:001 was not recovered in any EMJH semi-solid media.

When incubated at 37 °C, strain HB15B203 was only cultured in HAN semi-solid media. Dinger zones were observed by 2 weeks post-inoculation. Evidence of visible growth was detected in 3 of 12 replicates in HAN semi-solid media within 7 days post-inoculation, and was confirmed by dark-field microscopy. Strain HB15B203 was not detected by 4 weeks post-inoculation in either EMJH or T80/40/LH semi-solid media, Table 3 and Fig. 2.

**Figure 3:**
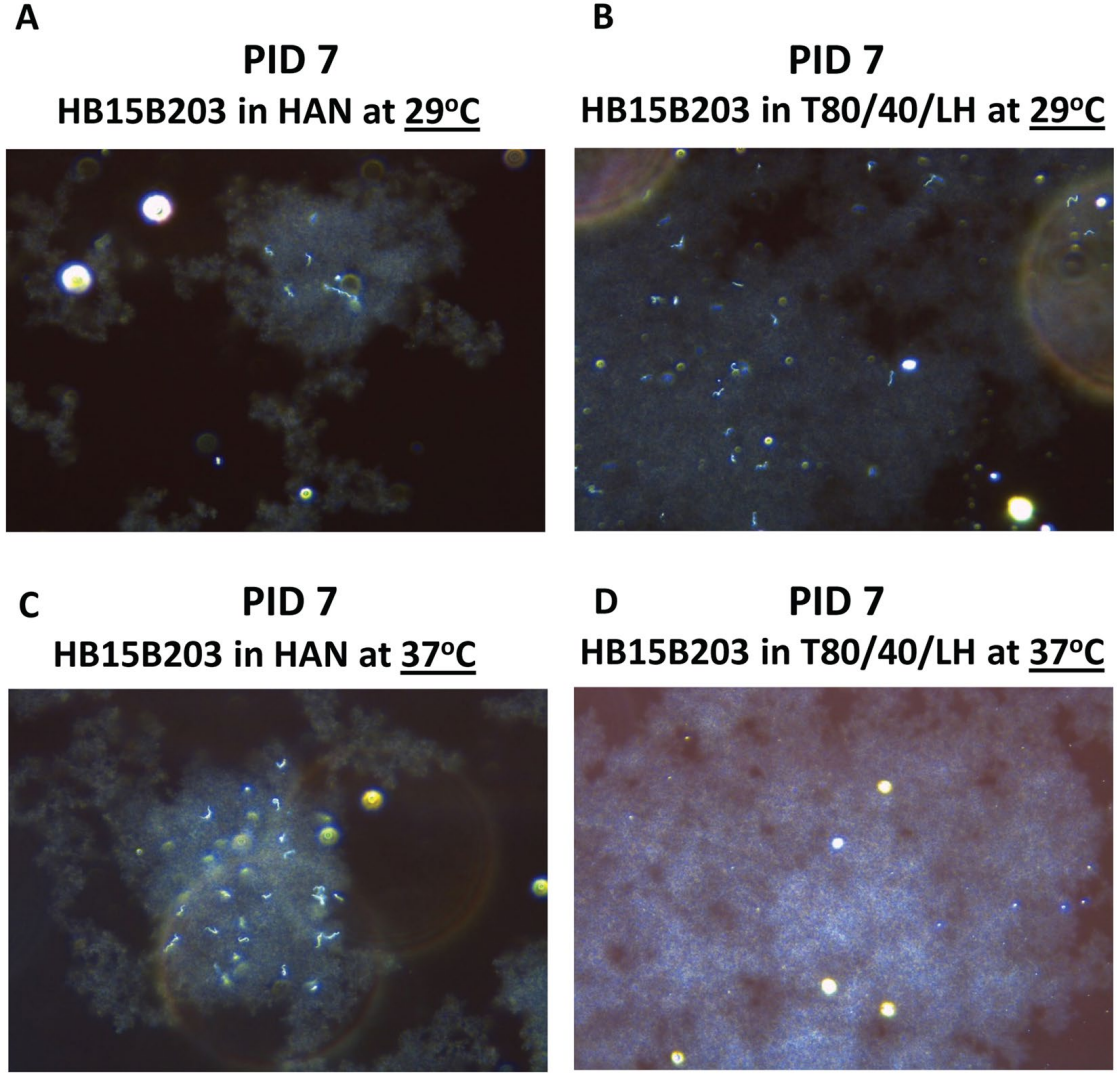
Images of semi-solid media after inoculation with water derived HB15B203 by dark-field microscopy. Seven days after inoculation of HAN (A & C) or T80/40/LH (B&D) semi-solid media, at 29 °C (A&B) or 37 °C (C&D), with 100 µl of water derived strain HB15B203, a 10 µl aliquot was examined by dark-field microscopy (final magnification 200×).

In order to emulate the culture of fastidious leptospires from an environmental source, strain HB15B203 was stored in autoclaved tap water at a density of 3 ×10^6^ leptospires/ml and stored at room temperature in darkness. After approximately 8 months, the sample was enumerated at a density of 2 ×10^6^ leptospires/ml and used to inoculate EMJH, T80/40/LH or HAN semi-solid media incubated at 29 or 37 °C. When incubated at 29 °C, strain HB15B203 was cultured in both T80/40/LH and HAN semi-solid media by 4 weeks post-inoculation as evidenced by the presence of a Dinger zone, Table 4. Evidence of visible growth, confirmed by dark-field microscopy, was detected in HAN semi-solid media within 2 weeks post-inoculation, whereas Dinger zones were observed in T80/40/LH semi-solid media at the same time point, Table 4. Though not detected visually, examination of semi-solid cultures with dark-field microscopy at 7 day post-inoculation confirmed the presence of leptospires, Fig. 3A,B.

**Table 4:** Growth of water derived cultures of HB15B203 in semi-solid media at 29 and 37 °C.

Strain	Medium @ 29 °C	PIW 1	PIW 2	PIW 3	PIW 4
HB15B203	EMJH	−	−	−	−
HB15B203	T80/40/LH	−	D	D	D
HB15B203	HAN	−	+	+	D
Strain	Medium @ 37 °C	PIW 1	PIW 2	PIW 3	PIW 4
HB15B203	EMJH	−	−	−	−
HB15B203	T80/40/LH	−	+	+	−
HB15B203	HAN	+	D	D	D

*L*. *borgpetersenii* strain HB15B203 was inoculated into EMJH, T80/40/LH or HAN semi-solid media incubated at 29 and 37 °C. Cultures were visually assessed for growth at post-inoculation week (PIW) 1, 2, 3 and 4. Cultures were scored as having no visible growth (-), visible growth confirmed by dark-field microscopy (+) or a visible Dinger zone (D) confirmed by dark-field microscopy. Results are derived from four replicates of culture (N = 4).

When incubated at 37 °C, strain HB15B203 was cultured in both T80/40/LH and HAN semi-solid media. Visible growth was observed by 7 days post-inoculation in HAN media which progressed to a Dinger zone by 2 weeks, Table 4 and Fig. 3C. No visible growth was observed by 7 days in T80/40/LH media, Fig. 3D but was detected at 2 and 3 weeks post-inoculation. However, growth had collapsed by 4 weeks post-inoculation and leptospires were not detected by dark-field microscopy, Table 4. EMJH semi-solid media did not support growth of HB15B203 derived from long term storage in water at 29 °C or 37 °C by 4 weeks post-inoculation.

### Liquid cultures

Three days after inoculation of media, cultures derived from kidney samples were examined and enumerated for the presence of intact motile leptospires by dark-field microscopy.

**Figure 4:**
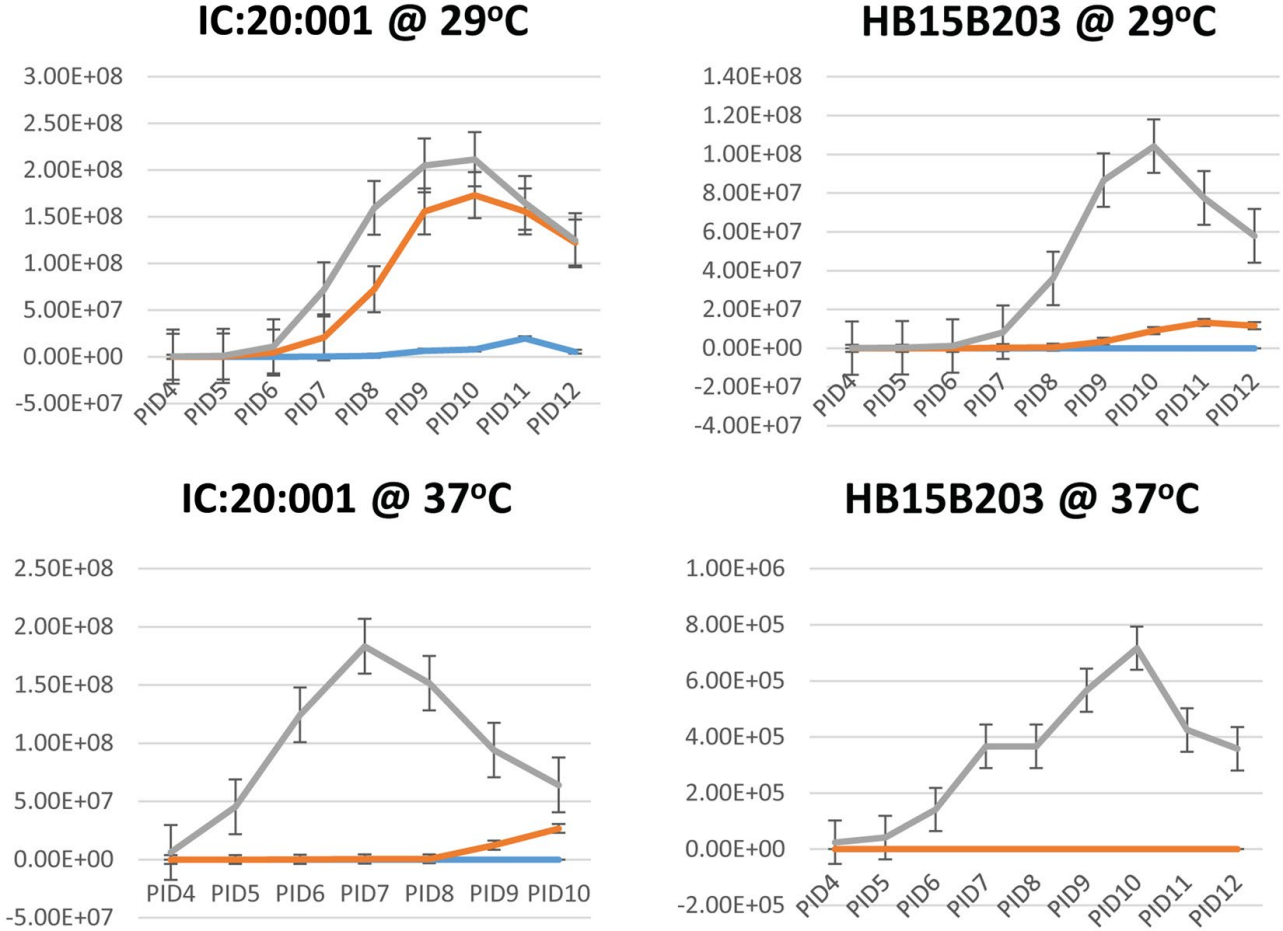
Growth curves of kidney derived cultures of IC:20:001 and HB15B203 incubated at 29 °C and 37 °C. *L*. *interrogans* strain IC:20:001 and *L*. *borgpetersenii* strain HB15B203 were enumerated by dark-field microscopy after inoculation into EMJH (blue), T80/40/LH (Orange) or HAN (Grey) media and incubation at 29 °C or 37 °C. Growth curves are averages of triplicate cultures from four hamsters (N = 12). Standard error bars are shown. Post-inoculation day (PID) of media is indicated on the X-axis and numbers of leptospires/ml of media are indicated on the Y-axis.

When incubated at 29 °C in liquid T80/40/LH and HAN, strain IC:20:001 reached a maximum density of ~1.5–2 ×10^8^ leptospires/ml at ~10 PID (post-inoculation days), Fig. 4. A maximum density of ~2 ×10^7^ leptospires/ml was observed for strain IC:20:001 in EMJH liquid media at PID 11. When incubated at 29 °C in liquid HAN, strain HB15B203 reached a maximum density of ~1 ×10^8^ leptospires/ml at 10 PID while the maximum density observed in T80/40/LH was ~1.3 ×10^7^ at 11 PID. Strain HB15B203 was detected in liquid EMJH media incubated at 29 °C, though cell density did not exceed 10^5^/ml.

When incubated at 37 °C in liquid HAN, strain IC:20:001 reached a maximum density of 1.8 ×10^8^/ml at 7 PID. By PID 9, strain IC:20:001 was at a density of ~1.2 ×10^7^ in T80/40/LH which increased to ~2.7 ×10^7^ on PID 10 but no growth was observed in EMJH by the same time point. When incubated at 37 °C in liquid HAN, strain HB15B203 reached a maximum density of ~7 ×10^5^ leptospires/ml at 10 PID. HB15B203 was not detected in T80/40/LH or EMJH incubated at 37 °C at any time point.

**Figure 5:**
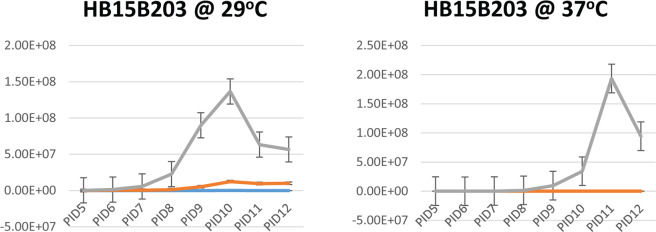
Growth curves of water derived cultures of HB15B203 at 29 °C and 37 °C. *L*. *borgpetersenii* serovar Hardjo strain HB15B203 were enumerated by dark-field microscopy after inoculation into EMJH (blue), T80/40/LH (Orange) or HAN (Grey) media and incubation at 29 °C or 37 °C. Growth curves are averages of triplicate cultures. Standard error bars are shown. Post -inoculation day (PID) of media is indicated on the X-axis and numbers of leptospires/ml of media are indicated on the Y-axis.

EMJH, T80/40/LH and HAN liquid media were also inoculated with strain HB15B203 which had been stored in autoclaved tap water for ~8 months. By 10 PID, HB15B203 reached a maximum density of ~1×10^8^/ml in HAN when incubated at 29 °C compared to T80/40/LH which reached ~1.2 ×10^7^ leptospires/ml at the same time point, Fig. 5. When incubated at 37 °C, strain HB15B203 was only detected in HAN, and at a maximum density of ~1.4 ×10^8^/ml on PID 10.

### Serotyping of cultures

A cultured isolate from each of the two different groups of experimentally infected hamsters was selected for confirmation of serotype. An isolate cultured from hamsters infected with IC:20:001 had a titer of 1:3200 when tested with reference antiserum against serovar Icterohaemorrhagiae, and no reactivity when tested with reference antiserum against serovar Hardjo. Conversely, an isolate cultured from hamsters infected with HB15B203 had a titer of 1:3200 when tested with reference antiserum against serovar Hardjo, and no reactivity when tested with reference antiserum against serovar Icterohaemorrhagiae.

## Discussion

Leptospires are chemoorganotrophic bacteria that require long chain fatty acids as an absolute requirement^17^. Serovar Canicola and serovar Pomona could not synthesize long chain fatty acids de novo, nor elongate fatty acids chains^18^. However, stearate could be degraded to palmitate and saturated acids were desaturated to form monounsaturated fatty acids. However, such pioneering studies on the metabolic requirements of leptospires for fatty acids were often based on a limited number of serotypes, and for which genetic information had yet to be obtained. Thus, investigators continued to evaluate multiple media types and supplements. It is routine to evaluate multiple sources and lots of bovine serum albumin (BSA) to determine which lot will support the growth and maintenance of in-house strains before committing to purchase of larger volumes. Thus, BSA can act as an essential exogenous supply of fatty acids and nutrients, especially for highly fastidious strains of leptospires.

New species and serovars of pathogenic leptospires continue to be isolated from reservoir hosts of leptospirosis^19,20^ and the application of selective antimicrobial agents (STAFF) has facilitated the isolation of several new species of leptospires from soils^21^. However, reliance on commercially available EMJH as the only media type available to culture serovars of leptospires circulating in animal and environmental samples is biased for the recovery of isolates to only those serovars and species that are supported by EMJH, and not those that require additional and alternative growth factors. Hence, the goal of this work is to provide an alternative media, HAN, using chemically defined components that could support the growth of pathogenic leptospires directly from animal tissue. In order to make comparisons between media as rigorous as possible in a laboratory setting that would emulate the culture of kidney from a reservoir host of infection, HAN was evaluated in direct comparison to EMJH and T80/40/LH for its ability to support the growth of two divergent species and serovars of pathogenic leptospires directly from animal tissue; *L*. *interrogans* serovar Copenhageni strain IC:20:001 and the more fastidious *L*. *borgpetersenii* serovar Hardjo strain HB15B203.

Optimal growth temperature for leptospires is 28–30 °C in semisolid (0.1–0.2%) agar media, with a generation time of 6–16 h, although many primary pathogenic isolates may grow slower^17^. Growth of leptospires in semi-solid media is characterized by an opaque subsurface “Dinger” zone of growth^22,23^. EMJH supported the growth of strain IC:20:001 directly from the kidney of an experimentally infected hamster in semi-solid media incubated at 29 °C, and within 2 weeks. Similarly, T80/40/LH and HAN were also positive but evidence of growth was apparent within 7 days indicating a shorter lag time. Both T80/40/LH and HAN also supported the growth of strain HB15B203 within 2 weeks but no growth was observed in EMJH, even after 4 weeks. These results highlight the fastidious nature of strain HB15B203 relative to strain IC:20:001, but perhaps more importantly, reflect a lack of growth factors in EMJH that are present in T80/40/LH and HAN that are required to support the growth of *L*. *borgpetersenii* serovar Hardjo strain HB15B203, a bovine isolate of *Leptospira*.

An optimal growth temperature of 28–30 °C for pathogenic leptospires is counterintuitive given its ability to infect, and persist in, a range of mammalian hosts including domestic animal species whose body temperature can range from ~37–39 °C^24^. Though EMJH has been used to grow pathogenic leptospires at 37 °C, such studies typically utilize strains that are well adapted to *in vitro* growth. EMJH does not support the primary isolation of leptospires at 37 °C and while T80/40/LH was able to support growth of strain IC:20:001 at 37 °C in some cases by 3 weeks, no growth of HB15B203 was detected by 4 weeks, Table 3. In contrast, Dinger zones were detected in HAN semi-solid media incubated at 37 °C within 2 weeks, Fig. 2.

Both T80/40/LH and HAN semi-solid media supported the growth of strain HB15B203 that had been stored in autoclaved tap water for at least 8 months. However, when cultures were incubated at 29 °C, Dinger zones were readily detected by 2 weeks in T80/40/LH semi-solid whereas they were not detectable in HAN media until 4 weeks. In contrast, when incubated at 37 °C, Dinger zones were readily detected by 2 weeks in HAN but not in T80/40/LH. Collectively, these results indicate that the success of isolating leptospires is directly related to the sample source i.e. animal versus environmental, as well as incubation temperature and components in the media. Leptospires cultured from water had a shorter lag phase using T80/40/LH while leptospires cultured from kidney had a shorter lag phase with HAN; this result suggests that growth factors in HAN more adequately support the metabolic requirements of *in vivo* derived leptospires at 37 °C.

Though the use of semi-solid media is considered optimal for the primary isolation of leptospires^17^, the use of all three media types were evaluated without the presence of agar. Both T80/40/LH and HAN supported the growth of strain IC:20:001 at 29 °C whereas only HAN supported logarithmic growth of strain HB15B203. At 37 °C, HAN also supported the growth of both strains but the highest density reached for strain HB15B203 peaked at day 10 at ~7 ×10^5^ leptospires/ml. It will be interesting to determine what factors are required to replenish the media in this case to allow sustained logarithmic growth. When cultured from autoclaved tap water, strain HB15B203 reached a density of 1 or 1.4 ×10^8^ leptospires/ml in HAN when incubated at either 29 or 37 °C, respectively.

All the components in HAN are completely chemically defined, except for the BSA. The choice of salts, vitamin B_12_, Tween 80, glycerol and sodium pyruvate are based on previously published formulations^13,25,26^. The specific choice of hemin (Sigma 9039) as a source of Fe was based on preliminary comparisons using different sources of hemin and FeSO_4_ to support enhanced growth of pathogenic *Leptospira* (data not shown). Similarly, the specific choice of DMEM/Ham's Nutrient mixture F12 (Sigma 51448 C) was based on preliminary screening on different DMEM Nutrient mixtures and combinations thereof (data not shown). Additional modifications that may further enhance HAN include alternative sources of long chain fatty acids; T80/40/LH not only uses Tween 80 as a source of oleic acid, but Tween 40 as a source of palmitic acid and as required to support the growth of serovar Hardjo from cattle^16^. As presented, HAN does not have a source of palmitic acid so it remains to be determined if the inclusion of Tween 40 would provide added benefit. The exclusion of serum as a component in HAN at this stage is deliberate to avoid lot to lot variation and thus facilitate continued evaluation and optimization of individual HAN media components. However, supplementation with serum, as routinely used for EMJH, might be beneficial. The added benefit of incubating cultures in the presence of 1–5% CO_2_ also needs to be addressed^27,28^. Finally, a rigorous evaluation to more completely chemically define multiple sources of BSA, and its fatty acids fractions, is required to identify correlations between the presence/absence of fatty acids in BSA preparations that support/inhibit growth of specific serovars/species compared to others.

The advent of genomics has resulted in significant advances in our understanding of pathogenic mechanisms of leptospirosis, as well as redefining the taxonomy^11^. The genome of *L*. *borgpetersenii* is ~700 kb smaller than that of *L*. *interrogans* leading to the hypothesis that *L*. *borgpetersenii* is evolving toward a strict host-to-host transmission cycle with loss of gene function being associated with impairment of environmental sensing and metabolite transport and utilization^29^. A smaller genome might explain the more fastidious nature of serovar Hardjo but the use of T80/40/LH and HAN compensates for this since it is more effective than that of EMJH. An alternative explanation for our limited ability for the routine primary isolation of leptospires is our collective failure to understand the function of a large number of coding genes that are still annotated as hypothetical or predicted, and with as yet undefined functions, and which may play significant roles in the transport and metabolism of growth factors. The growth of leptospires in different media provides an opportunity to address these deficiencies. Significant advances have also been made in our understanding that leptospires modify gene and protein (and their respective post-translational modifications) expression in response to environmental cues, including those encountered during host infection^8,30–36^. It has long been hypothesized that leptospires cultured in media that more closely aligns with that encountered during infection, e.g. temperature, osmolarity, iron concentration, and serum concentration, would express gene, protein and virulence profiles more similar to those expressed by leptospires during infection^30,37–41^. The ability of HAN to support growth directly at 37 °C not only questions the dogma that optimal growth of leptospires occurs at 28–30 °C, but provides for alternative methods to prepare bacterin vaccines that may be more effective. Indeed, Matsunaga *et al*. have previously demonstrated that culturing leptospires in the presence of MEM to modify osmolarity resulted in increased expression of the virulence factors LigA and LigB^38^. Further, the ability to bypass the use of semi-solid media and go directly to liquid allows for the continued selection and evaluation of host derived isolates that can be used to seed larger volumes for the preparation and evaluation of bacterin vaccines.

Our evaluation of HAN is limited to two different strains that were selected to encompass representative isolates that in our hands are considered relatively “easy” (strain IC:20:001) and “difficult” (strain HB15B203) to grow in a background where both isolates cause a persistent renal colonization in small rodents. Kidneys from experimentally infected hamsters were harvested for culture at 3 weeks post-infection to emulate as closely as possible the naturally occurring renal colonization. The observed lag time of 5–7 days in our liquid cultures reflects the presence of leptospires in each inoculum below the routine level of detection by dark-field microscopy. It remains to be determined how well HAN will support the growth of other serovars and species of leptospires, particularly during primary isolation. It is expected that a biological sample from an incidental host that may contain larger numbers of leptospires in an initial inoculum would have a shorter lag phase which makes the use of culture from an incidental host promising as a diagnostic tool, and compatible with resources in diagnostic laboratories that may only have 37 °C incubators available. Limited studies in our laboratory using urine samples from experimentally infected rats^42^ suggest this is possible and is an area of active investigation. *L*. *interrogans* includes hundreds of serovars but serovar Copenhageni is the leading cause of acute disease in people. The ability to recover serovar Copenhageni from a patient’s samples within days highlights its potential for diagnostics. Additional applications for HAN include its use in agar plates to support the more rapid growth and selection of clonal isolates, or other experimental processes that require plating e.g. the selection of genetic mutants.

In 1974, Richard Ham highlighted the nutritional requirements of primary [mammalian] cultures as a neglected problem of modern biology^43^. His conclusions at that time continue to be especially relevant to the leptospirosis field in that we “cannot yet regard primary cultures as efficient research tools”. The study of animal and human leptospirosis continues to be a neglected area of research^44^. Renewed efforts are required to further optimize nutritional requirements for the primary isolation of the large and diverse range of species and serovars within the genus *Leptospira* that are circulating within domestic and wild animal populations.

## Methods

### Bacteria

*Leptospira borgpetersenii* serogroup Sejroe serovar Hardjo type bovis strain HB15B203 was isolated from a 10 year old dairy cow from Kansas^45^. *L*. *interrogans* serogroup Icterohaemorrhagiae serovar Copenhageni strain IC:20:001 was isolated from a rat kidney in Detroit, Michigan^46^. Prior to experimental infection of hamsters, strain HB15B203 was propagated in semi-solid T80/40/LH and strain IC:20:001 was propagated in semi-solid EMJH, at 29 °C.

### Media

EMJH (Ellinghausen-McCullough-Johnson-Harris) medium was prepared by adding 100 ml of *Leptospira* Enrichment EMJH to 900 ml *Leptospira* Medium Base EMJH (BD Difco, Sparks, MD, USA), supplemented with 5-Fluoruracil (5-FU, 100ug/ml, Sigma F-6627). T80/40/LH media was prepared as previously described^16^ with the following modifications: 5-FU was used at 100 µg/ml and Nalidixic acid was not used. HAN media was prepared as follows: 10 g of Bovine Serum Albumin (BSA, Probumin Vaccine Grade, Millipore, Lot 107) was added to 500 ml water and slowly stirred until completely dissolved. The following stock solutions were added: thiamine chloride (1 ml, 0.5 g/100 ml water, Sigma T1270), calcium chloride (1 ml, 3.0 g/100 ml water, BDH 100703), magnesium chloride (1 ml, 3.0 g/100 ml water, BDH 101494), zinc sulphate (1 ml, 0.4 g/100 ml water, BDH 102994), hemin (10 ml, 6.52 mg/100 ml water, Sigma 9039), vitamin B_12_ (1 ml, 0.02 g/100 ml water, Sigma V6629), Tween 80 (12.5 ml, 10 ml/100 ml water, Sigma P8074), glycerol (1 ml, 10% solution in water, BDH 101184), and ammonium chloride (1 ml, 25% solution in water, BDH 100173). The following salts were added: disodium phosphate (1.0 g, BDH 102494), monopotassium phosphate (0.3 g, BDH 102034), sodium chloride (1.0 g, BDH 102414), and sodium pyruvate (0.1 g, Sigma P5280). To this solution, 200 ml Dulbecco’s Modified Eagle’s Medium/Ham’s Nutrient Mixture F12 (Sigma 51448 C) and 5-Fluoruracil (100 µg/ml, Sigma F-6627) was added. The solution was stirred for one hour, pH adjusted to 7.4 using sterile filtered 1 N NaOH, and final volume adjusted to one liter; this was then filter sterilized using a 0.22 µm bottle top filter. T80/40/LH and HAN media were made with the same batch of BSA. All media was stored in the dark at room temperature until use. HAN media has a shelf life of at least 6 months when stored at room temperature in the dark.

All three media formulations were also prepared with 0.15% Noble agar to use as semi-solid media. Filter sterilized transport medium comprised BSA (10 g), monopotassium phosphate (87 mg) and sodium phosphate dibasic (664 mg) per liter, pH 7.4. In all cases, media was prepared using autoclaved sterile-filtered bioreagent water (Sigma W3500).

### Experimental design

All animal infections were approved by the National Animal Disease Center’s Institutional Animal Care and Use Committee (ARS-2018–745) in accordance with the Guide for the Care and Use of Laboratory Animals and/or the Guide for the Care and Use of Agricultural Animals in Agricultural Research and Teaching. In order to generate infected animal tissue to evaluate each media type for the primary isolation of pathogenic leptospires, groups (N = 4) of Golden Syrian Hamsters (Envigo) were inoculated by intraperitoneal injection with either ~10^7^ of strain IC:20:001 or ~10^8^ of strain HB15B203 in a final volume of 0.5 ml. A separate control group was inoculated with negative growth media. Animals were weighed daily, Supplementary Fig. 1, and samples collected 21 days post infection. In order to emulate the culture of fastidious leptospires from an environmental source, strain HB15B203 was stored in autoclaved tap water for 8 months. Prior to storage, HB15B203 was propagated at 37 °C in HAN liquid medium to a density of 1 ×10^8^/ml. Ten milliliters of culture was harvested by centrifugation at 10,000 *g* for 30 min at 4 °C. The pellet was discarded and the supernatant was transferred to a new tube and centrifuged again using the same conditions. The supernatant was then discarded and the remaining pellet resuspended in 10 ml autoclaved tap water; the density of leptospires was 3 ×10^6^/ml. The tube was placed in the dark, at room temperature for 8 months.

### Culture

Kidneys were harvested using aseptic technique. One half of each kidney from a single animal was added to 9 ml transport medium and macerated. One ml of this suspension was diluted in another 9 ml aliquot of transport medium. Of this, 100 µl was used to inoculate 6 ml of indicated semi-solid medium, or 200 µl was used to inoculate 10 ml of indicated liquid media. Three replicates of each media, per strain, per hamster kidney were performed. All cultures were prepared in duplicate so that they could be incubated at both 29 °C and 37 °C.

Strain HB15B203 was stored in autoclaved tap water for 8 months. At this time, 100 µl was used to inoculate 6 ml of indicated semi-solid medium, or 200 µl was used to inoculate 10 ml of indicated liquid media. All cultures were prepared in duplicate so that they could be incubated at both 29 °C and 37 °C. The virulence of water derived leptospires after 8 months was confirmed since two of four hamsters that received an intraperitoneal inoculation of 0.5 ml (containing 1 ×10^6^ leptospires) were kidney culture positive 3 weeks later (data not shown).

### Assessment of growth of leptospires

Liquid cultures were examined directly by dark-field microscopy to assess for viability and motility. Intact motile leptospires were counted by dark-field microscopy, as previously described^47^. Growth in semi-solid media was assessed visually and scored as having no visible growth (-), visible growth which was confirmed by dark-field microscopy (+) or the presence of a Dinger zone confirmed by dark-field microscopy (D).

### Microscopic agglutination test (MAT)

The MAT was performed on sera from experimentally infected hamsters according to OIE guidelines^48^. The serogroup of cultured isolates was validated with reference antisera obtained from the Leptospirosis Reference Laboratory at the National Veterinary Services Laboratory, USDA (Ames, IA).

## Supplementary information

Supplemental information.
