# Supplementary material for: Isolation and propagation of leptospires at 37 °C directly from the mammalian host
Source: Sci Rep. 2020 Jun 15;10:9620. doi: 10.1038/s41598-020-66526-4 (PMC7296004; doi:10.1038/s41598-020-66526-4)
Supplement: Supplementary file 1 — Supplemental information. [file 41598_2020_66526_MOESM1_ESM.docx]

**Isolation and propagation of leptospires at 37^o^C directly from the mammalian host.**

Richard L. Hornsby, David P. Alt & Jarlath E. Nally*.

Infectious Bacterial Diseases Research Unit, National Animal Disease Center, Agricultural Research Service, United States Department of Agriculture, Ames, IA, U.S.A.

*Corresponding author: [Jarlath.nally@usda.gov](mailto:Jarlath.nally@usda.gov)

**Supplementary Material**

**Supplementary Figure 1. Daily hamster weights.** Results are averages from 4 hamsters in each group inoculated with negative media (blue), ICO2001 (grey) or HB15B203 (orange). Standard error bars are indicated. Post-inoculation day (PID) is indicated on the X-axis and weight (g) is indicated on the Y-axis.

**
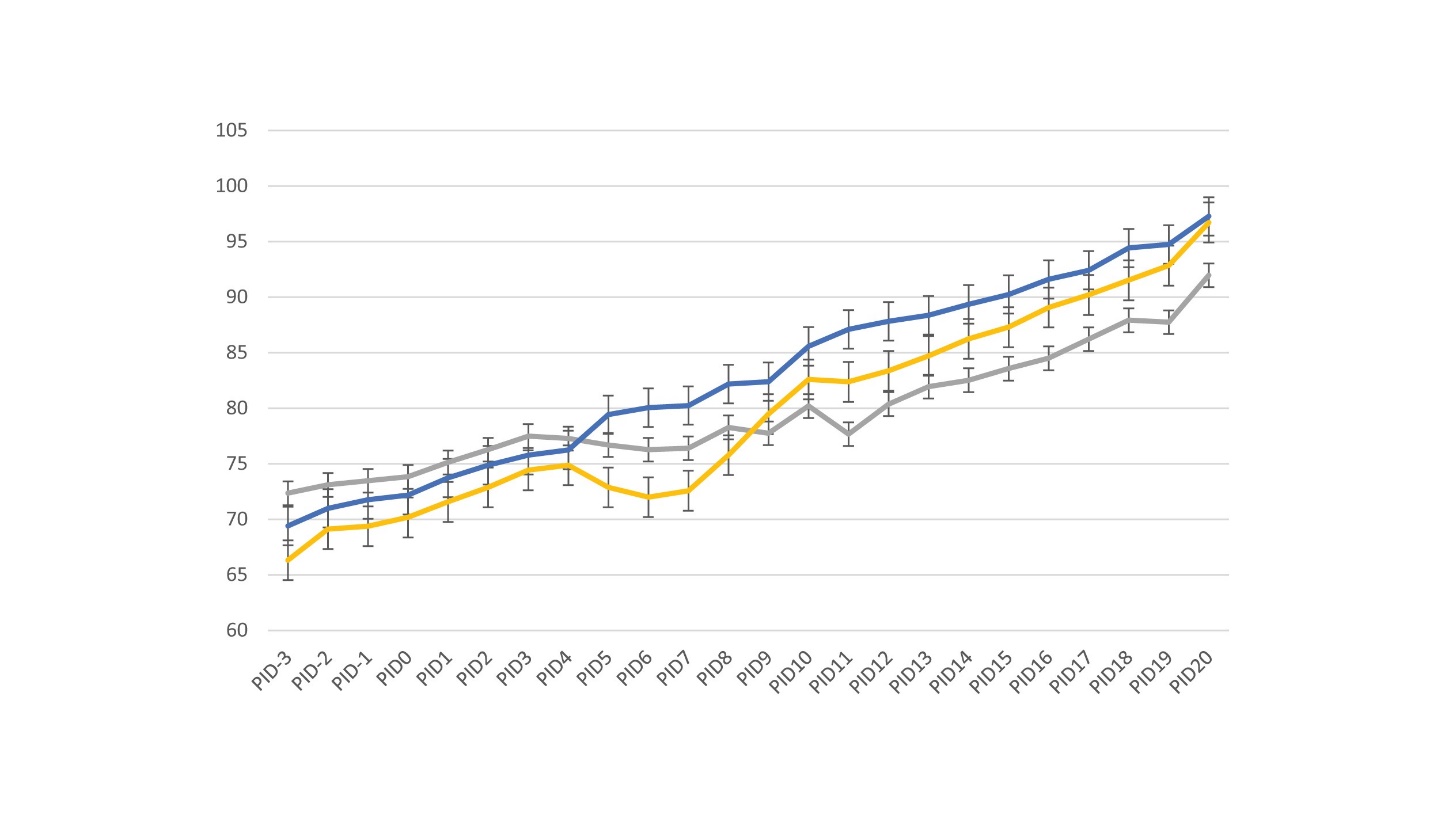
**
